# The Pathology of Orthopedic Implant Failure Is Mediated by Innate Immune System Cytokines

**DOI:** 10.1155/2014/185150

**Published:** 2014-05-07

**Authors:** Stefan Landgraeber, Marcus Jäger, Joshua J. Jacobs, Nadim James Hallab

**Affiliations:** ^1^Department of Orthopaedics, University Hospital Essen, University of Duisburg-Essen, Hufelandstraße 55, 45122 Essen, Germany; ^2^Department of Orthopedics, Rush University Medical Center, 1735 W Harrison MC107, Chicago, IL 60612, USA

## Abstract

All of the over 1 million total joint replacements implanted in the US each year are expected to eventually fail after 15–25 years of use, due to slow progressive subtle inflammation at the bone implant interface. This inflammatory disease state is caused by implant debris acting, primarily, on innate immune cells, that is, macrophages. This slow progressive pathological bone loss or “aseptic loosening” is a potentially life-threatening condition due to the serious complications in older people (>75 yrs) of total joint replacement revision surgery. In some people implant debris (particles and ions from metals) can influence the adaptive immune system as well, giving rise to the concept of metal sensitivity. However, a consensus of studies agrees that the dominant form of this response is due to innate reactivity by macrophages to implant debris where both danger (DAMP) and pathogen (PAMP) signalling elicit cytokine-based inflammatory responses. This paper discusses implant debris induced release of the cytokines and chemokines due to activation of the innate (and the adaptive) immune system and the subsequent formation of osteolysis. Different mechanisms of implant-debris reactivity related to the innate immune system are detailed, for example, danger signalling (e.g., IL-1*β*, IL-18, IL-33, etc.), toll-like receptor activation (e.g., IL-6, TNF-*α*, etc.), apoptosis (e.g., caspases 3–9), bone catabolism (e.g., TRAP5b), and hypoxia responses (Hif1-*α*). Cytokine-based clinical and basic science studies are in progress to provide diagnosis and therapeutic intervention strategies.

## 1. Introduction

Total hip and knee replacements are examples of successful surgical interventions with overall success rates of >90% at ten years after surgery [1]. However, increasing time after surgery correlates with greater incidence of loosened/failing hip and knee arthroplasties, where survival rates at 15–20 years after operation are very low <50%. Currently, 40,000 hip arthroplasties have to be revised each year in the US because of painful implant loosening and it is expected that the rates of revision will increase by 137% for total hip and 601% for total knee revisions over the next 25 years [2]. Painful loosening is a serious long-term complication because of the high clinical/surgical risks of revision surgery and the associated high health-care costs. The number of revisions is accompanied by diminishing bone stock and the need for even larger implants, which increases the risk profile. Improvements in surgical techniques, materials, and implant designs have reduced the problem over the years by reducing particle production but the underlying problem remains. Thus diagnosing and stopping debris induced osteolysis are particular problems that have not been solved and are needed to improve the long term performance of joint replacement implants.

Aseptic loosening (no infection) is the main cause for revision surgery over the mid- and long-term and is responsible for >70% of hip revisions and >44% of knee revisions [3, 4]. Various biomechanical factors like micromotion may play a role in the induction of aseptic loosening directly but also indirectly through the formation of additional wear particles. The various implant debris induced biological reactions have been well established as the central causal problem [5–7]. This local bone loss (or peri-implant osteolysis) is initiated by aseptic inflammatory responses to phagocytosis of small implant wear particles (generally <10 microns in diameter) resulting in increased proliferation and differentiation of osteoclast precursors into mature osteoclasts [8–10]. Various cytokines and chemokines are involved in this inflammatory activation of osteoclasts. This paper will discuss implant debris (e.g., wear particle) induced release of cytokines and chemokines due to activation of the innate and the adaptive immune system and the subsequent formation of osteolysis and how this knowledge is currently used for diagnosis and therapy.

## 2. Innate Immune System Response to Wear Debris Particles

### 2.1. Macrophages

**Figure 1:**
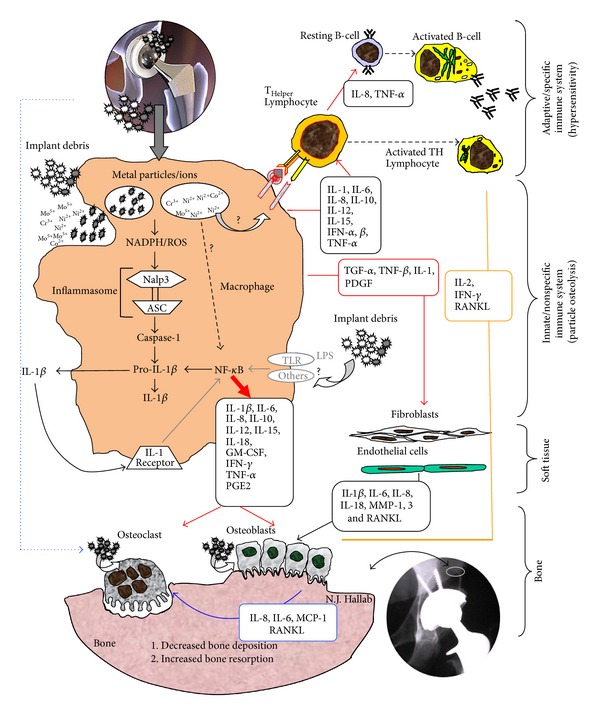
Schematic of how the inflammasome pathway is centrally involved in the pathology of implant debris-induced local cytokine responses (courtesy of Bioengineering Solutions Inc.).

Inflammatory responses to implant debris over time have been attributed to macrophage reactivity and have been the primary focus of investigation over the past 40 years. Recent studies demonstrate a predominance of M1 macrophages in response to implant debris challenge (released metal ions and particles), which produce primarily proinflammatory mediators that affect other local cell around implants (Figure 1) [11]. Thus, given that wear particles are biologically active and influence the innate immune pathway, the amount, appearance, rate of production, time of exposure, and antigenicity of the wear particulates are important [12, 13]. It has been shown that macrophages release a host of M1 associated cytokines after contact with wear debris. These include IL-1*α*, IL-1*β*, IL-6, IL-10, IL-11, IL-15, tumor necrosis factor *α*  (TNF-*α*), transforming growth factor *α*  (TGF-*α*), granulocyte-macrophage colony stimulating factor (GM-CSF), macrophage colony stimulating factor (M-CSF), platelet-derived growth factor, and epidermal growth factor (Figure 1) [14]. It is likely that more subtle, less studied cytokines and tissue responses are involved in this reactivity as well. The interaction of all these cytokines is very complex and not fully understood yet. While M-CSF and others activate the formation of osteoclasts directly, IL-1, TNF*α*, and IL-6 can affect osteoblasts and other cells which in turn activate osteoclasts and increase cytokine release by macrophages [14]. GM-CSF is responsible for formation of multinucleate giant cells (MNGCs), which act very similar to osteoclasts.

Chemokine expression by macrophages, fibroblasts, and osteoblasts exposed to implant debris is also a central innate immune effector reaction to implant debris [15–19]. The chemokines, particular to implant aseptic loosening pathology, include IL-8, MCP-1 MIP-1*α*, CCL17/TARC, and CCL22/MDC [20]. IL-8, a CXC chemokine, is upregulated by macrophages and MSCs in periprosthetic tissues by different types of wear particles like titanium, CoCr, and UMHWPE [21, 22]. This migration of macrophages and osteoclasts to the sites around implants leads to accelerated osteolysis [20].

Increased expression of MCP-1, MIP1 (CCL-2), and MIP 1*α* (CCL3) was observed in periprosthetic tissues from failed arthroplasties and also in macrophages analyzed cell culture after exposure to different types of wear particles [16]. In contrast to MIP1*α*, an increased release of MCP-1 was also observed from fibroblasts after exposure to titanium and PMMA particles [17]. Reactions in vivo to UHMWPE and PMMA particle challenge were judged responsible for recruitment of macrophages [23, 24] given systemic migration of macrophages in a mice model decreased when deficient in the CCR2 receptor [23] or after blocking CCR2 receptor [24]. Blocking CCR1 or CCR2 eliminated the migration of MSCs in vitro and blocking CCL17/TARC and CCL22/MDC in osteoclasts and hFOB and their cognate receptor CCR4 in osteoclasts precursors decreased recruitment of osteoclast precursors to the bone-implant interface [25] and are currently potential targets of future interventions [24, 26].

### 2.2. Bone Responses 

#### 2.2.1. Osteoclasts

The role of osteoclasts is central to osteolysis, as they are the primary bone resorbing cells. RANK(L) signalling is central for the activation of osteoclasts and activates a variety of downstream signalling pathways required for osteoclast development, but crosstalk with other signalling pathways also fine-tunes bone homeostasis both in normal physiology and disease [27, 28]. The degree to which other cells with the potential to resorb bone (e.g., macrophages) can participate directly in debris induced osteolysis is not known. The role of released cytokines such as TNF-*α* is also important, but their contribution to osteoclast formation is currently unclear.

Kadoya et al. showed that MNGCs express some markers which are also expressed by osteoclasts, like tartrate-resistant acid phosphatase (TRAP) and vitronectin receptor (VNR) [29]. This applied to MNGCs located on the bone side of the soft-interfacial-tissue (located between implants and bone) but not to those on the implant side. Additionally, in vitro studies have shown that macrophages, exposed to wear debris particles, are capable of a type of low-grade bone resorption [30]. But although if the bone resorbing activity of macrophages is very reasonable, given their abundance and close ontogenic relationship with osteoclasts, it is far from certain that macrophages participate in bone destruction and further studies will be necessary to clarify their role in this context.

Osteoclasts in turn are also capable of phagocytosing a wide size range of ceramic, polymeric, and metallic wear particles. After particle phagocytosis, they remain fully functional, hormone responsive, bone resorbing cells [31, 32], thus showing that at least in vitro there is substantial plasticity between these key cell types involved in implant associated osteolysis that derive from the same precursor cells in bone marrow. Even participation of the early forms of macrophages and osteoclasts, mesenchymal stem cells, have been implicated in aseptic loosening [21], where the endocytosis of wear particles reduced proliferation and osteogenic differentiation and induces an increased production of IL-8 [21]. The association between MNGC and osteoclast formation does not reflect some sort of transdifferentiation or plasticity, but rather than that all macrophage populations include immature macrophages that form both osteoclasts and mature macrophages. This makes it difficult to distinguish MNGC from osteoclasts in histological sections unless they are opposed to the bone surface.

#### 2.2.2. Osteoblasts

Osteoblasts are stimulated by wear particles to produce the osteoclastogenesis factors RANKL and M-CSF [33] and cytokines such as IL-6 and IL-8 [34]. The same study also reports a slightly increased expression of VEGF induced by all particle entities and decreased de novo synthesis of type 1 collagen as well as increased expression of matrix metalloproteinase (MMP)-1.

### 2.3. Soft Tissue Responses

#### 2.3.1. Fibroblasts

Soft tissue cells such as fibroblasts are also actively involved in osteoclastogenesis and bone resorption [35]. The most prominent upregulated genes and proteins secreted by fibroblasts in response to wear debris were matrix metalloproteinase 1 (MMP-1), monocyte chemotactic protein-1 (MCP-1), IL-1*β*, IL-6, IL-8, cyclooxygenase 1 (cox-1), cox-2, leukemia inhibitory factor, transforming growth factor beta 1 (TGF*β*1), and TGF*β* receptor type I. Stimulated fibroblasts express RANKL and osteoprotegerin.

### 2.4. Adaptive Immune Responses

#### 2.4.1. Lymphocytes

**Figure 2:**
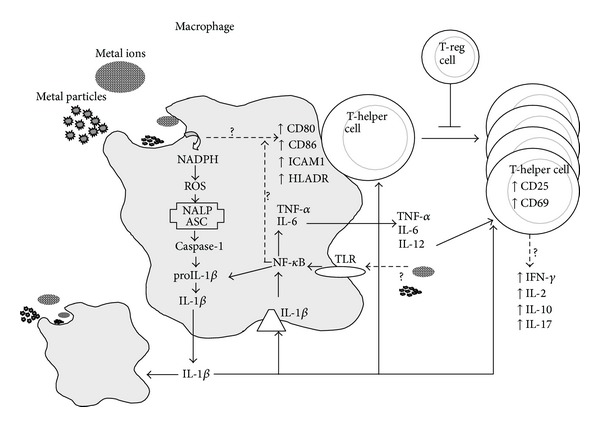
Innate immune system (i.e., macrophage) interactions with implant debris produce danger signalling (inflammasome) and pathogen (NF-*κ*B) associated cytokines such as IL-1*β* and TNF*α* and increased expression of costimulatory molecules such as CD80/86, ICAM1, and HLADR. These innate responses can trigger adaptive immune responses where destructive TH1 type cytokine profiles require T-regulatory cells (e.g., IL-10) to control this response (courtesy of Bioengineering Solutions Inc.).

Lymphocytes can play a crucial role in the peri-implant “debris-reactivity” environment as well. It is well recognized that T and B lymphocytes are present in peri-implant tissues [36, 37]. The subtypes of T cells that dominate implant debris associated responses are T-helper (TH) and not T cytotoxic/suppressor (TC/S) which have been found at an in vivo ratio of 7.2 : 1 [38]. Of the T-helper cells present, TH1 cells predominate as characterized by production of IFN-*γ* and IL-2 and to a lesser degree IL-17, fractalkine, and CD40, which indicate the possibility of TH17 activity (versus nonobserved TH2 cell mediated IL-10 responses) [39, 40]. The involvement of specific lymphocyte responses TH1 cells that can also recruit and activate macrophages, with relatively very few participating local cells, suggests that the role of adaptive immune response may be overlooked and falsely (in some cases) attributed to innate macrophage innate nonspecific immune responses, Figure 2. It has been difficult to readily identify these responses in peri-implant tissues, by such signature cytokines as IL-2, interferon-*γ*, TNF-*α*, and IL-2 receptors [41]. But some studies using mRNA detection instead of tissue immunohistochemistry (IL-2) have shown the increased expression of these TH1 cytokines [42, 43]. Furthermore, macrophages and lymphocytes seem to interact with each other via lesser reported coreceptors and cytokines such as IL-15 and its related IL15 receptor (IL-15R*α*) on the macrophages, respectively, IL2 receptor (IL-2R*β*) on the lymphocytes [44]. These TH responses have been characterized as type IV delayed type hypersensitivity. DTH response to metal implant debris is an adaptive slow cell mediated type of response. Metal-antigen sensitized and activated DTH T-cells release various cytokines which recruit and activate macrophages, Figure 2 [38], such as IL-3 and GM-CSF (promotes hematopoiesis of granulocytes); monocyte chemotactic activating factor (MCAF) (promotes chemotaxis of monocytes toward areas of DTH activation); IFN-*γ* and TNF-*β* (produce a number of effects on local endothelial cells facilitating infiltration); and migration inhibitory factor (MIF) (signals macrophages to remain in the local area of the DTH reaction). Activated macrophages have increased ability to present class II MHC and IL-2 and can trigger the activation of more T-DTH cells, which in turn recruit/activate more macrophages, which recruit/activate more T-DTH cells, in a runaway cycle of inflammation, without T-regulatory cells (and other factors) to inhibit the response over time. A DTH self-perpetuating response can create extensive tissue damage. Forms of metal sensitivity testing such as lymphocyte transformation test (LTT) and patch testing (for skin reactions) are the only means to predict/diagnose those individuals that will have an excessive immune response to metal exposure that may lead to premature implant failure (approximately >1-2% patients/yr) [38].

## 3. Initial Mechanisms for the Wear Particle Related Activation of the Innate Immune System

**Figure 3:**
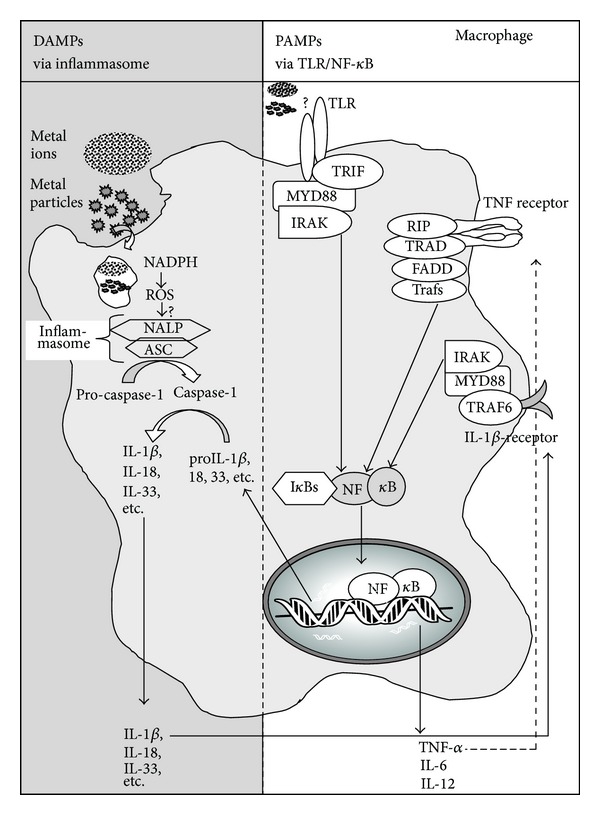
Schematic of intracellular innate immune responses to implant debris (metal ions and particles) that produce both DAMP and PAMP pathway activations through lysosomal destabilization (DAMPs) and either TLR or cytokine receptor activation (PAMPs), resulting in the collaborative interaction of the inflammasome and NF-*κ*B pathways (courtesy of Bioengineering Solutions Inc.).

Despite new understandings of implant related cytokine/chemokines networks that are their release by different peri-implant cell types, the mechanisms mediating cellular interaction with debris particles and the subsequent activation of macrophages to produce and release the inflammatory mediators remain incomplete. Past investigations have shown the importance of PAMPs (e.g., toll-like receptors, TLRs) in vivo, in the periprosthetic tissues of patients with aseptic loosening [45–47] and in TLR-knockout mouse models (MyD88 knockout mice) where lower amounts of cytokines and osteolysis were induced by polymethylmethacrylate (PMMA) implant debris particles than wild-type mice [20, 48]. The MyD88 dependent pathways of TLR signalling result in activation of nuclear factor NF-*κ*B, which has been long shown to play a role in particle induced osteolysis and the production of proinflammatory cytokines such as TNF*α*, IL-1*β*, and IL-12, Figure 3 ****[49].

Toxicity responses are another facet of innate immune activation where apoptosis and hypoxia responses have been found to be induced by implant debris [50–52]. Soluble and particulate metal debris have been shown to induce hypoxia-like pathology resulting in HIF-1*α* compensatory responses to metal implant debris by promoting both the induction of hypoxia (HIF-1*α*) and tissue angiogenesis (VEGF) providing a specific mechanism which explains why local soft tissue growths (fibrous pseudotumors) and apoptosis responses can form in some people with certain orthopedic implants [52]. The induction of apoptosis associated processes by implant debris has also been correlated with implant debris in vivo [53, 54]. And more recently ceramic and polyethylene implant debris particles have been shown to induce some form of apoptosis of macrophages in vitro [50, 51]. This in vitro evidence has been supported by in vivo immunohistochemistry of central apoptosis-related mediators such as caspase-3 associated with macrophages, giant cells, and T-lymphocytes in local tissues (capsules and interfacial membranes) of patients with aseptic hip implants [55, 56]. The importance of apoptosis associated mediators has been made clear by murine osteolysis models that demonstrated inhibition of apoptosis by a pan-caspase inhibitor leads to decreasing bone resorption by osteoclasts [57] and presumably decreased amounts of apoptosis associated cytokines like interleukin-8 (IL-8), monocyte chemoattractant protein-1 (MCP-1), intercellular adhesion molecule-1, and type-1 interferon [58, 59].

The influence of danger signalling, that is, inflammasome activation, is a relatively new approach in orthopedics. Nonpathogen derived stimuli typically activate immune cells through a danger signal pathways, the central components of which are termed the “inflammasome” [60]. Effective immune system activation requires specific receptors that recognize both pathogen associated molecular patterns (PAMPs) and danger associated molecular patterns (DAMPs) to initiate innate proinflammatory responses, Figures 1 and 3 ****[61, 62]. Nonpathogen derived danger signals are triggered by DAMPs such as UV light, particulate adjuvants present in modern vaccines [63, 64], and recently have been discovered to be activated by implant debris [65]. Typical particulate DAMPs induce lysosomal destabilization, which cause an increase in NADPH (nicotinamide adenine dinucleotide phosphate-oxidase) and an increase in reactive oxygen species (ROS). The release of these intracellular contents is sensed by specific members of the NLR family, such as NALP3 (NACHT-, LRR-, and pyrin domain-containing protein 3). NALP3 protein, in association with ASC (apoptosis-associated speck-like protein containing a CARD domain), forms the intracellular multiprotein complex, that is, the inflammasome complex [66, 67]. Activation of the inflammasome (NALPs-ASC complex) leads to the cleavage of pro-caspase-1 into active caspase-1 (previously known as ICE, interleukin-1 converting enzyme). Active Caspase-1 is required for the processing and subsequent release of active proinflammatory cytokines such as IL-1*β* and IL-18 (and others) by cleaving intracellular pro-IL-1*β*, pro-IL-18, and so forth into their mature forms, IL-1b and IL-18. As IL-1*β* is one of the main cytokines for activation of osteolysis, an involvement in aseptic loosening is obvious, as a recent study has shown less osteolysis in caspase-1 knockout mice [68].

It is well accepted that the inflammatory factors previously described here drive osteoclast formation through progenitor recruitment and RANKL induction; however, the detailed mechanics of how this occurs remains unknown. IL-1, for example, strongly stimulates osteolysis in many contexts but does not affect OC formation directly yet is a very weak inducer of RANKL in bone cells in vitro.

## 4. Therapy of Aseptic Loosening by Regulation of the Innate Immune Response

New biologic treatments addressing the pathology of aseptic implant loosening are currently under development and in clinical trials. Some cytokine inhibitors have been investigated using in vitro and in vivo animal models. Potential treatments include the following.

 AM630 is a selective inhibitor of cannabinoid receptor 2 that inhibits IL-1*β* and TNF-*α* [69].
 LY294002 is a specific inhibitor of PI3 K that suppresses the expression of TNF-*α* [70].
 Tetrazykline inhibits MMP-9 [71].
 Simvastatin decreases ERK1/2 a phosphorylated protein which is stimulated by wear particles and involved in cell signalling activation of macrophages [72].

None of the aforementioned cytokine regulating drugs have been tested in clinical trials, due to the serious side effects and risks associated with immunosuppressive medications. Other potential candidates (for clinical treatment) include drugs indicated for the treatment of rheumatoid arthritis and other inflammatory diseases, such as traditional nonsteroidal anti-inflammatory drugs (NSAIDs), selective cyclooxygenase (COX) inhibitors (e.g., celecoxib), tumor necrosis factor (TNF) antagonists (e.g., etanercept, infliximab, adalimumab), and interleukin-1 antagonists (e.g., anakinra) [73]. However many investigators remain concerned about the application of these drugs for this pathology due to the antianabolic effects of NSAIDs and COX-2 inhibitors, and the immunosuppressive effects of the anti-inflammatory drugs [73]. Newer drugs using small interfering RNA (siRNA) have shown promise in vivo where a mouse model demonstrated that local delivery of lentivirus-mediated TNF-*α* small interfering RNA (siRNA) resulted in less implant debris induced TNF-*α*, IL-1, and IL-6 and overall in a less associated inflammation [74].

Furthermore, without clinically validated early detection biomarkers of implant loosening, by the time patients presents with pain and radiological evidence of loosening the implant is mechanically loose, and the associated continuous micromotion acts to prevent reintegration even if implant debris associated inflammation-induced osteolysis is arrested [73]. Thus diagnosis of early stages of aseptic loosening is paramount and is the focus of much continued research. Other nonimmune related counter measures to implant debris induced osteolysis have also focused on enhancing bone responses in the face of inflammation. Although beyond the scope of this review, two noteworthy anti-bone-resorption (i.e., osteoclast inhibiting) bisphosphonates (Etidronate and Alendronate) are currently being evaluated for long-term therapy [75–78], although the embrittlement of bone and cases of early fracture have tempered these efforts.

## 5. Conclusion

The serious pathology of aseptic osteolysis around joint replacement implants is intimately dependent on cytokines and chemokines released by innate and adaptive immune reactions and local cells around implants. These types of debris-induced inflammation are dominated by innate immune cell (macrophages) secretion of TNF*α*, IL-1*β*, IL-6, and PGE2, which causes peri-implant bone resorption. Given the increasing number of people receiving orthopedic implants the issue of biologic reactivity is growing more prevalent. There is a growing need for more targeted approaches of diagnosis and early intervention of unwanted debris-induced inflammation. New understanding of how sterile nonpathogen implant debris causes immune activation and other local reaction continue to be discovered, such as the inflammasome “danger signalling” pathway [60], and the induction of hypoxia and apoptosis related reactivity [52, 55, 56, 79]. Consequently new therapies (such as anti-TNF-infliximab, anti-IL-1*β*, IL-1*β*-receptor-antagonist anakinra, etc.) are under current investigation as targeting measurement and pharmacologic interventions. New diagnostic testing modalities (e.g., cytokines, chemokines, bone metabolism markers, and lymphocyte testing, LTT) are under investigation as candidate early diagnostic measures of debris induced inflammation. Soon these studies will lead to early detection and thus treatment of debris induced inflammation leading to improved long term implant performance.
